# The epidemiology of lung cancer in Hungary based on the characteristics of patients diagnosed in 2018

**DOI:** 10.1038/s41598-024-70143-w

**Published:** 2024-08-29

**Authors:** István Kenessey, Petra Parrag, Mária Dobozi, István Szatmári, András Wéber, Péter Nagy, Csaba Polgár

**Affiliations:** 1 National Institute of Oncology and National Tumor Biology Laboratory, Budapest, Hungary; 2 Department of Pathology, Forensic and Insurance Medicine, Semmelweis University, Budapest, Hungary; 3 Schools of PhD Studies, Semmelweis University, Budapest, Hungary; 4 Cancer Surveillance Section, International Agency for Research On Cancer (IARC/WHO), Lyon, France; 5 Laboratory of Redox Biology Research Group, Department of Anatomy and Histology, HUN-REN–UVMB, University of Veterinary Medicine, Budapest, Hungary; 6 Chemistry Institute, University of Debrecen, Debrecen, Hungary; 7 Department of Oncology, Semmelweis University, Budapest, Hungary

**Keywords:** Incidence, Lung cancer in Hungary, Cancer registry, International comparison, Cancer, Cancer, Cancer epidemiology, Lung cancer

## Abstract

Among malignant diseases, lung cancer has one of the highest mortality and incidence. Most epidemiological studies conclude that Hungary faces the most severe burden in association with this disease. However, for various reasons estimates and population-based studies show discrepancies. In this study, an intense data cleansing was performed on lung cancer cases that were reported to the Hungarian National Cancer Registry in 2018, and the major clinico-pathological parameters as well as survival characteristics were described. Our population-based figures were compared to the European estimates. As a result of our thorough revision, the corrected incidence of lung cancer has fallen below the number of cases that were reported to the Registry from 11,746 to 9,519. We also demonstrate that Hungary did not show the highest incidence and mortality in Europe, but it is still among the ones with the worst raking countries, with 92.9 and 50.6 age standardized rate per 100 thousand capita among males and females, respectively. Analysis of the annually reported case numbers revealed a gender-specific difference in incidence trends: while from 2001 to 2019 it slightly decreased among males, it increased among females. The most dominant subtype was adenocarcinoma, which was more frequent among female patients. Unfortunately, most of the newly diagnosed cases were in advanced stage; thus, 5 year overall survival was 14.8%. We anticipate that in the longer term, a decrease in incidence and improvement in survival rates may be expected as a result of the development of primary and secondary prevention programs in the country.

## Introduction

According to the estimates of Global Cancer Observatory (GLOBOCAN), lung cancer showed the highest mortality and the second highest incidence among malignant diseases in 2020 worldwide^1^. Most epidemiological studies of the International Association for Research on Cancer (IARC) mentioned Hungary as the global leader of lung cancer with estimated 10.200 new cases and 9.000 deaths annually, which means approximately 50.1 and 42.4 age standardized rate per 100 thousand capita^2–4^. Additionally, the lung cancer cases reported annually to the Hungarian National Cancer Registry (HNCR) exceed the GLOBOCAN estimates by at least 20 percent. On the other hand, Hungarian death certificate-based mortality statistics are almost in line with the GLOBOCAN estimates, with the latter reporting 2.1 percent more cases for 2018. While the GLOBOCAN database is crucial for assessing cancer burden, both Hungarian lung cancer estimates suggest some discrepancies compared to the registered case numbers. Although the Hungarian National Cancer Registry is operating according to international guidelines, when calculating Hungarian incidence, GLOBOCAN did not apply these datasets. Instead, the reported Hungarian death numbers of the previous years were projected to the studied year, while calculation of incidence is based on the mortality-to-incidence ratio (MIR) of the neighboring countries^3^.

Although the HNCR has been operating since the 1950s, it was only transformed into a population-based registry in 1999, as the result of a ministerial decree. According to its regulations, HNCR now covers the entire domestic population (100%) of approximately 9.6 million people. In the recent years the HCNR has been operating with a team of 6 employees: 1 administrator, 1 statistician, 1 IT personnel, 2 post-docs and 1 PhD student. Since 2017, the reporting process has been exclusively web-based. Physician–patient encounters are registered in the hospital’s IT system, from which IT professionals filter the data and submit reports directly to the HNCR. During submission, built-in validation checks mark the most critical coding errors, which require correction and resubmission. However, HNCR staff are not capable to verify the reliability of the entire reported dataset. Hence, the HNCR database contains a significant proportion of uncertain cases, with only about 60 percent of the registered tumors verified through morphological examination^5^.

Undoubtedly, the high prevalence of smoking is associated with the significant burden of lung cancer in Hungary^6,7^. However, compared to neighboring countries cultural similarities and behaviors do not justify these extraordinary differences. Thus, considering the aforementioned inaccuracies, we have suspected methodological errors in the background of this discrepancy. Our previous analysis revealed that in the HNCR database only 60% of cases were verified by histological or cytological examination, which suggested significant proportion of miscoded entries^5^. Therefore, the aim of the recent evaluation was to provide a clearer picture about annual new lung cancer cases in Hungary, focusing on the year of 2018.

## Results

### Descriptive statistics

Originally, 11,746 patients were filtered out from the HNCR database with ‘C34’ ICD10 coded diagnosis in 2018. During our campaign, 9,519 patients were confirmed as true cases by the reporting hospitals (81%), in 2,206 cases the code of ‘lung cancer’ was found to be falsely used, while in another 21 cases the lung lesions remained to be considered ‘uncertain behavior’. Out of the confirmed cases, 58.2% was male, and 41.8% was female (Table 1). The most common age cohort among all patients was 60–69 years, with 82.5% of patients falling between the ages of 55 and 79. Among both sexes combined, 41.9% of confirmed lung cancer cases proved to be adenocarcinomas. The second most common subtype was squamous cell carcinoma at 20.6%, followed by small cell type at 10.3%, while the relatively rarer and miscellaneous types accounted for 7.3% of the cases. It must be noted that histology-based subtype allocation was absent in 19.9% of the cases. The most common stage at diagnosis was Stage IV, accounting for 35.1% of the cases, followed by Stage III at 13.2%, Stage II at 10%, and Stage I at 4.2%. Staging information was not available in 37.5% of the cases. According to the reported treatment database, surgery was performed in 3,327 cases (35%), chemotherapy was applied in 4,087 cases (42.9%), 2,704 patients were treated with radiotherapy (28.4%), immune check-point inhibitors were administered in 614 cases (6.5%), while 3,366 patients did not receive any curative oncotherapy (35.4%).

**Table 1:** General characteristics of patient group diagnosed with lung cancer (C34 ICD10 code) in Hungary in 2018.

	Total number of patients: 9519 (100%)
Sex	
male	5,540 (58.2%)
female	3,979 (41.8%)
Age cohorts	1 (0%)
20–24	4 (0%)
25–29	4 (0%)
30–34	23 (0.2%)
35–39	72 (0.8%)
40–44	179 (1.9%)
45–49	558 (5.9%)
50–54	1,015 (10.7%)
55–59	2,011 (21.1%)
60–64	2,151 (22.6%)
70–74	1,649 (17.3%)
75–79	1,029 (10.8%)
80–84	523 (5.5%)
85 +	300 (3.2%)
Histology type	
small cell carcinoma	980 (10.3%)
adenocarcinoma	3,991 (41.9%)
squamous cell carcinoma	1,960 (20.6%)
miscellaneous cell types	698 (7.3%)
NA	1,890 (19.9%)
Stage	
I	402 (4.2%)
II	955 (10%)
III	1,252 (13.2%)
IV	3,343 (35.1%)
NA	3,567 (37.5%)
Applied oncology treatment	
surgery	3,327 (35.0%)
chemotherapy	4,087 (42.9%)
irradiation	2,704 (28.4%)
immune checkpoint inhibitors	614 (6.5%)
none	3,366 (35.4%)

Gender specific analyses revealed that adenocarcinoma was the most common tumor among both sexes, its frequency was significantly higher in females than in males (59% vs 47.5%, respectively, P < 0.001). Squamous cell carcinoma was relatively more common among males compared to females (31.7% vs. 17.3%, respectively, P < 0.001). In addition, where staging information was available, more than half of the cases were classified as the most advanced stage, with a relative male predominance, while Stage I proved to be relatively more frequent among females (Table 2).

**Table 2:** Gender distribution of the main available clinico-pathology parameters of patient group diagnosed with lung cancer (C34 ICD10 code) in Hungary in 2018.

	Number of patients with available parameters (100%)		P
	male	female	
Age—median (min; max)	66 (20; 96)	67 (27; 100)	0.006 (Mann–Whitney)
Histology type			1.4 × 10^−43^ (Chi-square)
small cell carcinoma	537 (12.1%)	443 (13.9%)	
adenocarcinoma	2,110 (47.5%)	1,881 (59%)	
squamous cell carcinoma	1,407 (31.7%)	553 (17.3%)	
miscellaneous cell types	387 (8.7%)	311 (9.8%)	
Stage			2.1 × 10^−51^ (Chi-square)
I	176 (5.1%)	226 (9.1%)	
II	564 (16.3%)	391 (15.7%)	
III	749 (21.6%)	503 (20.2%)	
IV	1,971 (57.0%)	1,372 (55.1%)	

### The correction of Hungarian lung cancer annual incidence in 2018 based on cleansed database

**Figure 1:**
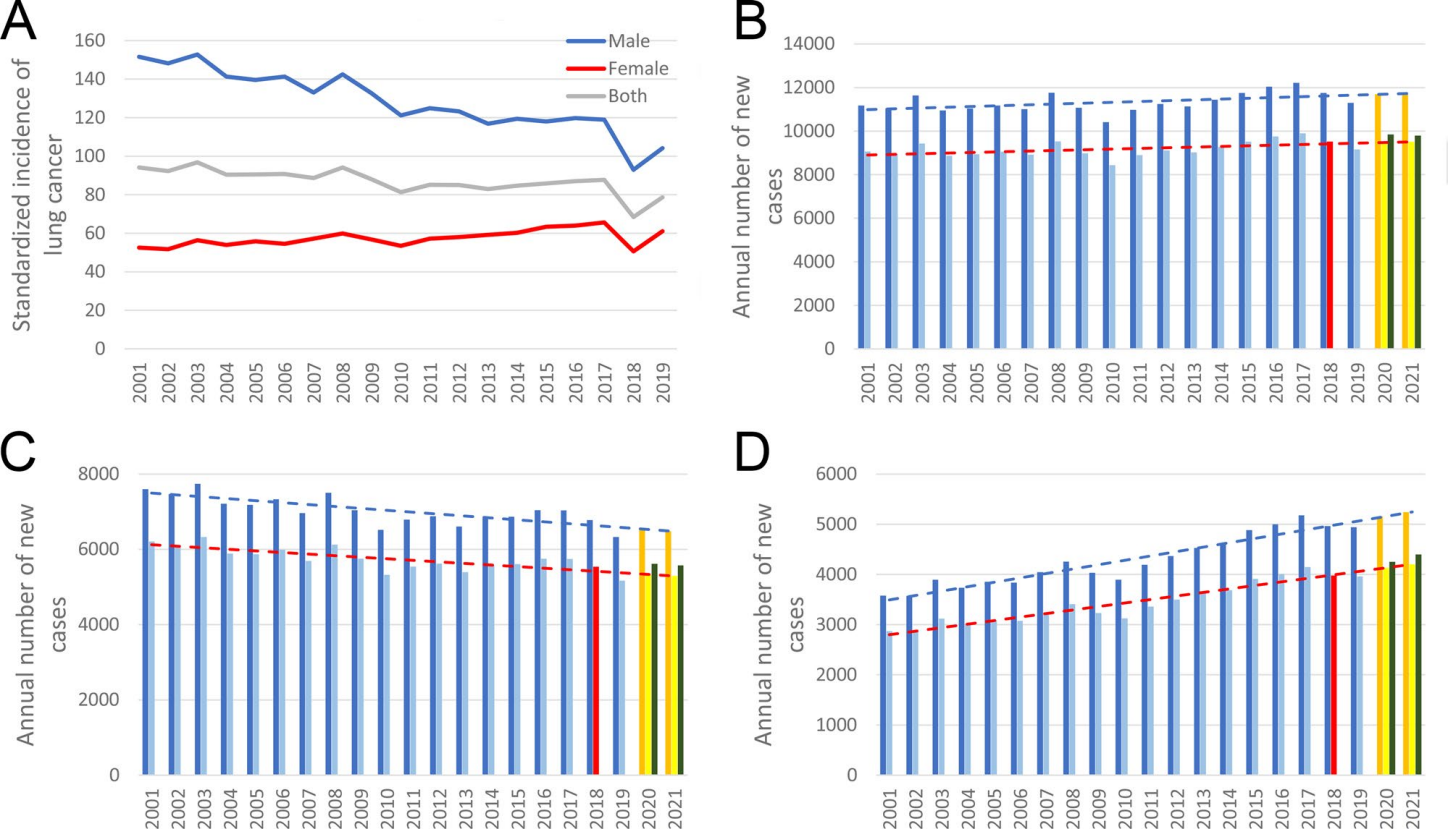
Correction of the reported annual number of lung cancer cases in Hungary according to the reference year of 2018. (**A**) Standardized reported case numbers according to European Standard Population 1976, except in case of 2018, where cleansed numbers are depicted. (**B**) Total number of new lung cancer cases by year. (**C**) Number of new lung cancer cases among males by year. (**D**) Number of new lung cancer cases among females by year. Dark blue columns and trend lines represent number of reported cases, light blue columns with red trend lines represent the real annual incidence based on the reference year 2018 (red columns). Orange columns mean prediction of the reported cases; yellow columns are for prediction of true lung cancer cases. The COVID-19 pandemic had an effect on the registration process, which resulted decreased reported numbers in 2020 and 2021 (green columns).

In line with international standards, where malignant cancers of the lung and trachea are categorized together, the reported C33 cases were incorporated into our cleansed database. Newly reported case numbers between 2001 and 2019 were standardized according to ESP1976 (Fig. 1A). In 2018, the standardized annual lung cancer incidence in Hungary was 68.4 per 100 thousand capita, with 92.9 per 100 thousand for males and 50.6 per 100 thousand for females. These values were significantly lower than those of other years based on the reports. Checking the number of reported C34 cases between 2001 and 2019, the numbers of reported annual new lung cancer cases were between 10,410 and 12,221 (Fig. 1B). Linear correction to the reference year of 2018 resulted a range between 8436 and 9904. Based on our linear regression analysis, the expected reported numbers of 2020 and 2021 were 11,695 and 11,732, respectively, which were corrected to 9,478 and 9,508 new cases. However, due to the obstruction effect of COVID19 pandemic, actual reported case numbers fell short of expectations, with 9842 new cases in 2020, and 9792 in 2021. Interestingly, those values were very close to our corrected hypothetical case numbers. Similar tendencies were observed when male and female patients were analyzed separately (Fig. 1C,D). Note that while incidence of lung cancer among males showed a slight decrease, among females a significant increase was observed.

### The survival of Hungarian lung cancer patients diagnosed in 2018.

**Figure 2:**
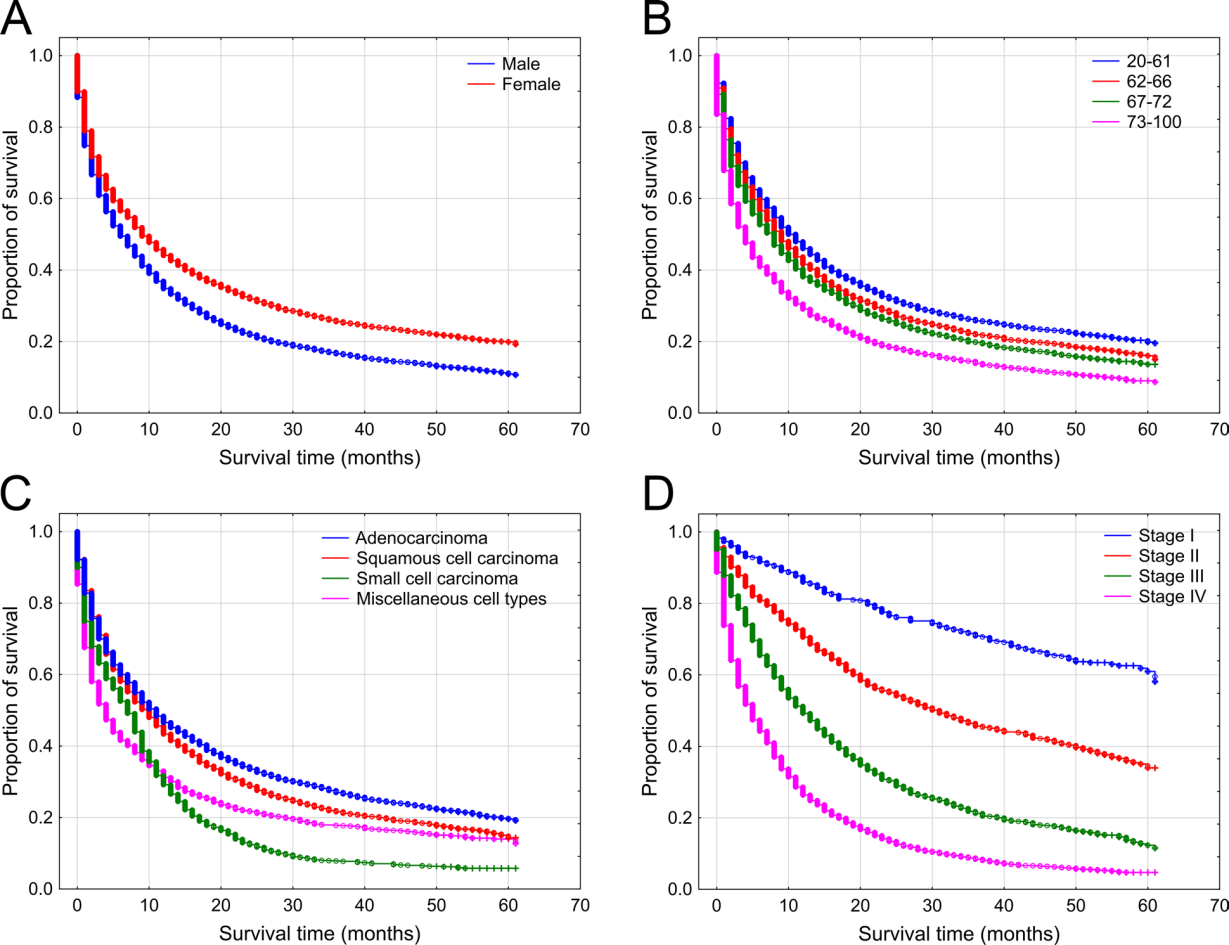
Survival analysis of Hungarian lung cancer cases diagnosed in 2018. Kaplan–Meier and log-rank analyses were performed by sex (**A**), age group quartiles (**B**), cell type (**C**) and cancer stage (**D**).

The 5 year overall survival of our lung cancer cohort was 14.8%. Kaplan–Meier and log-rank analyses revealed that female patients had significantly better overall survival compared to males, with median survival times of 9 months and 6 months, respectively (Fig. 2A). The Hungarian lung cancer patient group diagnosed in 2018 was categorized by age quartiles, and not surprisingly the youngest group showed the most favorable outcome, while the oldest group had the poorest survival (Fig. 2B). The median survival of 20–61 age group was 10 months, but this number in group 62–66 was 8 months, in group 67–72 it was 7 months, while in the oldest category, group 73–100 it proved to be only 3 months. In survival, significant difference was found among the different subtypes of lung cancers: adenocarcinoma cases were associated with the most favorable survival (median: 11 moths), followed by squamous cell carcinoma (median: 10 months), small cell carcinoma (median: 7 months), and miscellaneous cell types with a 4 month median (Fig. 2C). Of note, the latter category was formed due to statistical reasons, and it incorporated undifferentiated or unspecified tumors as well as relatively less common cell types such as neuroendocrine carcinoma or large cell cancer. Histology was available in 7629 cases (80.1%). Comparisons were also performed by cancer stage, which confirmed a long-known finding that more advanced cancer stages are associated with poorer outcomes (Fig. 2D). However, stage information was not available in 3567 cases (37.5%).

The Cox proportional regression model also confirmed that age, cellular type and stage are independent predictive parameters (Table 3). Regarding histology type, patients with small cell carcinoma were 34% (P = 0.001) more likely to die from lung cancer compared to the reference category of cellular type, namely the patients with adenocarcinoma. Following the same logic, patients diagnosed with squamous cell carcinoma had a 11% (P = 0.002) higher likelihood of dying, while those with miscellaneous cell type carcinoma were 57% (P < 0.001) more likely to die from lung cancer. Among the variables studied, stage proved to be the strongest predictor. Compared to the reference category of stage, namely Stage I, patients with Stage II lung cancer had more than twice (P < 0.001) the probability of dying, while those with Stage III lung cancer had over four times the probability of dying (P < 0.001) than those in the reference category. Lastly, Stage IV lung cancer patients were 4.78 times (P < 0.001) more likely to die from the disease than those having Stage I lung cancer.

**Table 3:** Cox proportional regression for the main parameters of confirmed Hungarian lung cancer cases diagnosed in 2018.

	RR (95% CI)	P
Age—median (min; max)	1.02 (1.016–1.023)	< 0.001
Histology type (reference: adenocarcinoma) small cell carcinoma squamous cell carcinoma miscellaneous cell types	1.342 (1.231–1.463) 1.116 (1.039–1.200) 1.570 (1.405–1.754)	0.001 0.002 < 0.001
Stage (Reference: I) II III IV	2.151 (1.786–2.592) 4.121 (3.446–4.927) 4.784 (4.144–5.523)	< 0.001 < 0.001 < 0.001

*RR* relative risk, CI confidence interval.

## Discussion

The most important risk factor of lung cancer is smoking^8,9^. Previous studies have identified Hungary as one of the countries with the highest prevalence of smoking, a factor strongly associated with a more severe epidemiological condition of tobacco-related diseases^6,10^. Given this context, the high incidence and mortality of lung cancer are not surprising, which is confirmed by both Hungarian population-based assessments and GLOBOCAN estimates^3,4,11^. Moreover, based on the registered case numbers of lung cancer, the trends of incidence followed the gender specific estimates of smoking: while among males, a slight decrease was found, in case of females, smoking and lung cancer incidence increased^6,12^. The drastic emergence of female lung cancer cases in Hungary was in concordance with international data and correlates well with the extent to which females adopted the habit of smoking and their vulnerability to the tobacco industry^13,14^.

On the other hand, each GLOBOCAN estimate considered Hungary as having exceptionally high incidence and mortality rates for lung cancer. However, smoking habits do not fully account for such extreme rates. In the HNCR database the low number of morphologically verified cases also suggests that the registered number of lung cancer cases surpasses the real number of patients^5^. This phenomenon may be partially explained by the irresponsible application of ICD10 codes^15^. The most common source of falsely reported lung cancer is the confusion between primary lung cancer and lung metastases. Additionally, when later examinations did not confirm the diagnosis of suspected malignant tumors, registration is not automatically capable of handling the elimination of false cases. Thus, unfortunately beside metastases, benign and non-neoplastic lesions are also present in the database. Additionally, the fact that most of the lung cancer cases were diagnosed in advanced stage with poor prognosis could mean that the actual proportion of death certificate only (DCO) cases may be high. Consequently, the failure to record DCO cases of entities with poor prognostic diseases like lung cancer may lead to the underestimations of the incidence.

**Figure 3:**
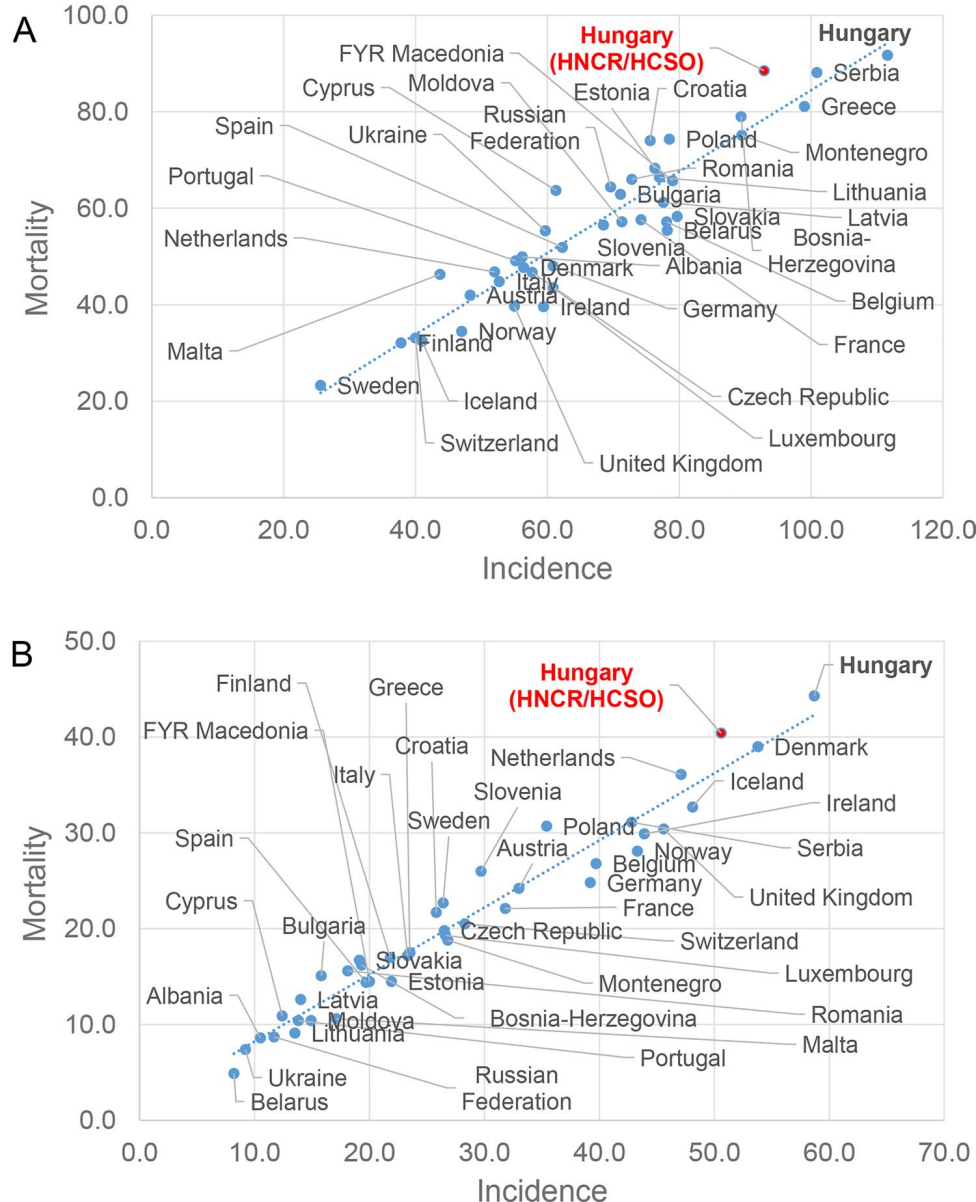
Comparison of standardized incidence and mortality of lung in 2018 by European countries according to the estimates of Ferlay et al. 2018 GLOBOCAN estimates Hungary as the highest incidence and mortality (black spots) among 40 European countries (blue spots) both in males (**A**) and females (**B**). Compared to the estimates, both the Hungarian Statistical Office and Hungarian Registry reported slightly lower case numbers (red spots). However, the epidemiology of lung cancer in Hungary is still among the highest burden countries.

Previous studies also attempted to receive a more exact picture about the Hungarian epidemiology of lung cancer^16–18^. The applied approach of Bogos et al. was very different from both population-based registration and estimates^16^. During their examinations, the financial database of the Hungarian health care system (NHIF) was taken as a basis, and a specific set of criteria determined whether a C34 coded case was accepted or rejected as a true lung cancer patient. Of note, Hungary has a unified health insurance system, which covers the whole population; therefore, when a health care service is applied, cancer cases appear in the financial database. Nevertheless, the rigorous criteria in the aforementioned studies potentially could have resulted in exclusion of some true cases. In this study, between 2011 and 2016 the annual incidence of lung cancer was calculated to be within the range of 6996 and 7158, and mortality was within 6045 and 6465. GLOBOCAN estimated the number of new lung cancer cases in 2012 and 2018 to be 9290 and 11,010, and the mortality to be 8070 and 8900, respectively^3,19^. In contrast to the hospital reported 10,500 and 12,000 new lung cancer patients, the recent study placed the range of true cases between 9000 and 10,000 which is larger than the numbers that were obtained from the financial database-based calculations, but slightly below the GLOBOCAN estimates. Putting our cleansed incidence and reported death numbers of lung cancer in an international context by comparing them with GLOBOCAN estimates of 2018, still places Hungary among the top countries within Europe (Fig. 3).

Regarding mortality, the Hungarian Statistical Office reported 8447 to 8896 annual death occurrences due to lung cancer between 2011 and 2019^11,20^. The comparison of measured incidence (9519) and mortality (8716) of lung cancer in 2018 revealed 91.5% for MIR, which exceeds GLOBOCAN’s global estimates of 84.8%. This fact suggests that a thorough revision of the mortality database is in order too, because these statistics might also contain numerous falsely coded lung cancer cases. This was examined in our previous paper^21^.

According to international epidemiological data, about 10 to 15% of all lung cancers are small cell cancers, which correlates well with our patient group^22,23^. However, we measured a relatively higher incidence of adenocarcinoma, particularly among females, with its proportion exceeding 40 percent, compared to the internationally published values which are around 30 percent^9^. 75 percent of our cases were discovered at advanced stage (III + IV), which is worse than in the US, but better than in India^24,25^. Importantly, similar to Hungary, population-based studies of Denmark, Sweden and Switzerland have not found an overall survival benefit for Stage IV disease, even with the introduction of new treatment options such as EGFR, ALK and ROS1 inhibitors^26–28^. This relative contradiction may be solved with a more stratified analysis of the Hungarian patients with Stage IV lung adenocarcinoma, focusing the applied agents; however, this task would exceed the current study. On the other hand, contrary to trends observed in the developed world, the incidence of Stage IV lung cancer cases in Hungary has increased over the past few decades, while the proportion of early-stage disease, where the greatest potential benefit from oncotherapy is expected, has remained unchanged^26^. Hence, according to our database, approximately 35% of the cases did not receive curative treatment, which rate is comparable with the magnitude of Stage IV cases. Our measured 5-year overall survival was worse compared to the result of the Hungarian longitudinal assessment, which was based on the aforementioned financial database^29^. However, the two studies agree in reported gender-specific difference was with more favorable outcome in female patients. Furthermore, our previous population-based survival analysis found that the efficacy of lung cancer treatment did not change reasonably from the years of 2000s to the 2010s.

Importantly, alongside the traditional treatments such as surgery, chemotherapy and irradiation, immune checkpoint inhibitors (ICIs) were introduced as a new therapeutic option for lung cancer at the end of 2010s^30^. Inhibitors of PD1 (such as nivolumab and pembrolizumab) and PD-L1 (such as atezolizumab) opened new possibilities for patients with advanced lung cancer, by reactivating the body’s own immune system which recognizes and destroys cancer cells^31^. Among our 9519 validated cases, 614 patients received at least one of the aforementioned agents, with 248 of these patients being diagnosed at Stage IV. Albeit our preliminary results suggested the survival benefit of immunotherapy compared to the outcome of lung cancer patients without ICIs (data not shown), to avoid potential bias deeper evaluations would be required with the adjustment of age, gender, histological subtype. In addition, the current database of the HNCR does not contain numerous important parameters that strongly determinates the prognosis (e.g. laboratory parameters such as lactate dehydrogenase, albumin, molecular subtype, general condition of the patients, accompanying diseases)^32,33^.

In summary, we believe our study provides a reasonable descriptive statistics of lung cancer in Hungary. We confirmed that in parallel with the shift of smoking habits, the annual incidence of males has either not changed significantly or slightly decreased over the past 20 years, while the incidence of females has increased. Despite the applied data adjustment, the lung cancer burden in Hungary is among the most severe in Europe. Consistent with the work of Gredner et al. that illustrated the comprehensive implementation of tobacco control policies^34^, survival progress is anticipated with the advancement of primary and secondary prevention efforts, which are reducing the prevalence of smoking, and enhancement of lung cancer screening activity.

## Materials and methods

### The HNCR database and data cleansing

Based on the International Classification of Diseases and Related Health Problems 10th Revision (ICD-10), the HNCR collects data of patients diagnosed with C00-C96 (malignant diseases), D00-D09 (in situ diseases), D30.3 or D33.0^35^. The HNCR covers the whole country, and according to the regulation, reporting of cases is mandatory. Nowadays, 143 hospitals report quarterly. Separate submission of clinical and pathological reports is required to a web-based application exclusively. Identification of personal data is based on the application of unique health insurance personal ID (= ’TAJ’), which allows to link records from different sources. Additionally, the HNCR receives death occurrence reports from the National Health Insurance Fund of Hungary (NHIF), which provides financial base for the whole domestic health care system. All patients were extracted from HNCR’s main database, where C34 (primary malignant neoplasm of the lung) was registered with the discovery date of 2018. Those cases where morphological examination (record from pathology) confirmed clinical diagnosis without other coded primary malignancy, were accepted as true lung cancer patients. Uncertain lung cancer cases were reported back to the data provider hospitals to confirm or reject their original diagnosis.

After data cleansing, main clinico-pathological parameters of the remaining lung cancer patients (e.g. age, gender, histological type, cancer stage, treatment type, date of diagnosis, date of death or last follow-up, alive or death status) were sorted. The simplified coding of TNM was based on UICC’s TNM Classification of Malignant Tumours, 8th edition^36^ as follows: Stage I-T1N0M0, T2aN0M0; Stage II-T2bN0M0, T1a-cN1M0, T2a-bN1M0, T3N0M0, Stage III.: T1a-cN2M0, T2a-bN2M0, T3N1M0, T3N2M0, T4N0M0, T4N1M0, T4N2M0, any T, N3M0; Stage IV—any T, any N, M1. Laterality was handled according to the recommendations of IARC, thus, where both lungs were affected with the same histology, only one case was counted^37^. Although the institutions were able to report the correct histology code according to ICD-O-3, during our analysis, subtypes were simplified as small cell cancer, adenocarcinoma, squamous cell cancer and miscellaneous type^38^. Despite the absence of histology, cases were accepted for the database if clinical diagnosis was later confirmed; however, further analysis did not calculate with that parameter. Treatment types were recorded using binary codes to indicate whether surgery, irradiation, or pharmacotherapy was administered. Falsely registered C34 codes were removed from the HNCR database as well as from our further analysis. The proportion of false and true cases was compared to the annual aggregated data from other years, which allowed reconsideration of incidence in the period between 2001 and 2020.

### Statistical analysis

Mortality data were publicly available from the Hungarian Statistical Office. Mortality and validated new case numbers were standardized according to the 1976 European Standard Population (ESP1976)^39^. Standardized incidence and mortality data were expressed as per 100 000 capita, which allowed direct comparison with the European estimates of GLOBOCAN for 2018^3^. Categorical parameters were analyzed and differences statistically tested using the Chi-square test.

Overall survival was determined as the time period from the diagnosis to the date of the last visit or death. The occurrence of death was reported by the patient’s care hospital, or by NHIF in cases where death occurred outside the hospital. Last follow-up date was 28th of February in 2023. Survival analyses were carried out using the Kaplan–Meier method and log-rank test statistics. Multivariate analyses of prognostic factors were performed using the Cox’s regression model. Differences were considered statistically significant when the p-value was lower than 0.05. The expected number of reported and true lung cancer cases were calculated by a linear regression model. Statistical calculations were performed by IBM SPSS Statistics v24 (Chicago, IL), Stata13 (College Station, TX), Statistica 14.0.1.25 (TIBCO Software, PaloAlto, CA) and RStudio 2021.09.1 (The R Project for Statistical Computing, Vienna, Austria).

### Ethical permission

Personalized data collection to the National Cancer Registry is mandated by a decree of the Hungarian Ministry of Human Capacities. Data handling was conducted in compliance with the General Data Protection Regulation (GDPR) of the European Union (EU). Data cleansing and statistical analyses were performed on a pseudonymized database. Since the Hungarian Cancer Registry was founded to perform these tasks, further ethical permission was not required.

## Strengths and limitations of the study

Albeit we believe that the recent study has provided a reasonable incidence dataset of lung cancer in Hungary, it has to be noted that it was based exclusively on data of a single year. The correction of reports from other years were based on the estimation that registration reliability was similar to that of the year 2018. Due to limited human resource, data cleansing and administration took time; therefore, more current data are available regarding lung cancer epidemiology. However, our results may validate GLOBOCAN2018 estimates. Our survival analysis did not incorporate cause-specific data, which means that the survival pattern represents overall survival. Although the HNCR receives casual mortality information with personal IDs, the Registry does not register DCO cases. On the other hand, according to the regulations, the performed autopsies should be reported. Furthermore, although the therapy of lung cancer is affected by the molecular subtype, the HNCR does not receive this parameter. The effectiveness of the different treatment modalities was also not evaluated.

## Data Availability

The datasets analyzed during the current study are not publicly available, since database contains personal information, but those are available from the corresponding author on reasonable request.
